# Characteristics of e-Cigarette Use Behaviors Among US Youth, 2020

**DOI:** 10.1001/jamanetworkopen.2021.11336

**Published:** 2021-06-07

**Authors:** Teresa W. Wang, Andrea S. Gentzke, Linda J. Neff, Emily V. Glidden, Ahmed Jamal, Eunice Park-Lee, Chunfeng Ren, Karen A. Cullen, Brian A. King, Karen A. Hacker

**Affiliations:** 1 Office on Smoking and Health, Centers for Disease Control and Prevention, Atlanta, Georgia; 2 Center for Tobacco Products, Food and Drug Administration, Silver Spring, Maryland; 3 National Center for Chronic Disease Prevention and Health Promotion, Centers for Disease Control and Prevention, Atlanta, Georgia

## Abstract

**Importance:**

Comprehensive surveillance of e-cigarette use behaviors among youth is important for informing strategies to address this public health epidemic.

**Objective:**

To characterize e-cigarette use behaviors among US youth in 2020.

**Design, Setting, and Participants:**

The 2020 National Youth Tobacco Survey, a nationally representative, cross-sectional, school-based survey of middle school (grades 6-8) and high school (grades 9-12) students, was conducted from January 16, 2020, to March 16, 2020. A total of 14 531 students from 180 schools participated in the 2020 survey, yielding a corresponding student-level participation rate of 87.4% and school-level participation rate of 49.9%. The overall response rate, a product of the school-level and student-level participation rates, was 43.6%.

**Exposures:**

Current (past 30-day) e-cigarette use.

**Main Outcomes and Measures:**

Self-reported current e-cigarette use behaviors (frequency of use, usual e-cigarette brand, and access source) by school level and flavored e-cigarette use and flavor types among current e-cigarette users by school level and device type. Prevalence estimates were weighted to account for the complex survey design.

**Results:**

Overall, 14 531 students completed the survey, including 7330 female students and 7133 male students with self-reported grade level and sex. In 2020, 19.6% (95% CI, 17.2%-22.2%) of high school students and 4.7% (95% CI, 3.6%-6.0%) of middle school students reported current e-cigarette use. Among them, 38.9% (95% CI, 35.2%-42.6%) of high school users and 20.0% (95% CI, 16.0%-24.8%) of middle school users reported e-cigarette use on 20 to 30 days within the past 30 days. Among current users, JUUL was the most commonly reported usual brand (high school: 25.4%; 95% CI, 18.8%-33.4%; middle school: 35.1%; 95% CI, 27.9%-43.1%). Among current users, the most common source of obtaining e-cigarettes was from a friend (high school: 57.1%; 95% CI, 52.6%-61.4%; middle school: 58.9%; 95% CI, 51.4%-66.1%). Among current users, 84.7% (95% CI, 82.2%-86.9%) of high school students and 73.9% (95% CI, 66.9%-79.8%) of middle school students reported flavored e-cigarette use. Fruit-flavored e-cigarettes were the most commonly reported flavor among current exclusive e-cigarette users of prefilled pods or cartridges (67.3%; 95% CI, 60.9%-73.0%), disposable e-cigarettes (85.8%; 95% CI, 79.8%-90.3%), and tank-based devices (82.7%; 95% CI, 68.9%-91.1%), followed by mint-flavored e-cigarettes.

**Conclusions and Relevance:**

These results suggest that although current e-cigarette use decreased during 2019 to 2020, overall prevalence, frequent use, and flavored e-cigarette use remained high. Continued actions are warranted to prevent and reduce e-cigarette use among US youth.

## Key Points

**Question:**

What are the characteristics of current (past 30-day) e-cigarette use among US high school and middle school students in 2020?

**Findings:**

This survey study of 14 531 students found that among the 19.6% of high school students and 4.7% of middle school students who currently used e-cigarettes in 2020, 38.9% of high school users and 20.0% of middle school users self-reported use on 20 to 30 days during the past 30 days, and most reported flavored e-cigarette use.

**Meaning:**

These results suggest that although current e-cigarette use among youth significantly decreased during 2019 to 2020, use prevalence, frequent use, and flavored e-cigarette use remained high.

## Teaser

This survey study characterizes e-cigarette use behaviors among US youth in 2020 using data from the 2020 National Youth Tobacco Survey.

## Introduction

During the past decade, e-cigarette use has increased considerably among US youth; this trend was first documented during 2011 to 2012, when the prevalence of current (past 30-day) e-cigarette use increased from 1.5% to 2.8% among high school (HS) students and 0.6% to 1.1% among middle school (MS) students.^1^ In 2014, the prevalence of current e-cigarette use among HS and MS students surpassed that of cigarette smoking, establishing e-cigarettes as the most commonly used tobacco product among US youth.^2^ Despite a subsequent decrease in e-cigarette use among youth during 2015 to 2016,^3^ a 78% relative increase in current e-cigarette use among HS students (11.7% to 20.8%) and a 48% relative increase among MS students (3.3% to 4.9%) occurred during 2017 to 2018,^4,5^ leading the US Surgeon General to declare e-cigarette use among youth an epidemic.^6^ In 2019, an estimated 27.5% of HS students and 10.5% of MS students reported current e-cigarette use.^7,8^

Multiple factors have contributed to the surge in e-cigarette use among youth, including e-cigarette advertising exposure, the availability of youth-appealing flavors, and the introduction of easily concealable devices with high nicotine content into the US marketplace.^9,10,11^ Although earlier versions of e-cigarettes were disposable and resembled conventional cigarettes, these products have since diversified to include rechargeable cartridge-based e-cigarettes known as pod mods. These newer versions of e-cigarettes^12,13^ typically deliver nicotine in the form of nicotine salts, instead of the free-base nicotine used in older e-cigarettes and conventional tobacco products, making it easier to inhale higher levels of nicotine with less irritation.^7,14^ Youth use of e-cigarettes is a public health concern, given that nicotine is a highly addictive substance that can harm adolescent brain development and prime the brain for addiction to other drugs.^6,9,10,11^

As the tobacco product landscape continues to evolve, sustained and timely surveillance of youth tobacco product use can play a role in guiding strategies to prevent and reduce youth tobacco product use, including use of e-cigarettes. Although estimates of the current prevalence of e-cigarette use among youth from the 2020 National Youth Tobacco Survey (NYTS) have been released,^15,16^ related data about e-cigarette use behaviors among youth remain to be examined. This study presents nuanced findings related to current e-cigarette use among HS and MS students, including frequency of use, usual brand, access sources, and patterns of flavored e-cigarette use among current e-cigarette users.

## Methods

### Data Source

The NYTS is a cross-sectional, school-based, self-administered survey of US MS (grades 6-8) and HS (grades 9-12) students. A stratified, 3-stage cluster sample design is used to produce a nationally representative sample of students in grades 6 to 12 who attend public and private schools. Participation in the NYTS was voluntary. Schools use passive or active parental permission forms at their discretion. Active parental permission was obtained via written form, but forms were collected and maintained by the teacher to maintain student privacy. For schools that elected to use passive permission forms, parents signed and returned the permission form only if they did not want their child to participate in the survey. Written student assent was not collected on any form to maintain privacy; however, students were told the survey was voluntary, so assent was assumed for students who continued to take the survey. All data were deidentified. The NYTS study protocol was approved by the institutional review board of the US Centers for Disease Control and Prevention, and this study follows the American Association for Public Opinion Research (AAPOR) reporting guideline. Detailed information about NYTS sampling design and recruitment procedures is available online.^17^

Data collection for the 2020 NYTS occurred from January 16, 2020, to March 16, 2020. Survey administration was originally planned to extend through May 15, 2020, but data collection was truncated because of widespread school closures related to the COVID-19 pandemic. A total of 14 531 students from 180 schools participated in the 2020 NYTS, yielding a corresponding student-level participation rate of 87.4% and school-level participation rate of 49.9%. The overall response rate, a product of the school-level and student-level participation rates, was 43.6%. Participants completed the survey within a single class period using electronic tablets with a programmed survey application featuring skip patterns. Nonbranded tobacco product images and descriptions with example brands were displayed at the beginning of each tobacco product–specific section. Absent students and whole classes unavailable on the day of survey administration could participate in make-up surveys using a web-based version of the questionnaire. Race and ethnicity were self-reported; students could select 1 or more of the following fixed categories for race: American Indian or Alaska Native, Asian, Black or African American, Native Hawaiian or other Pacific Islander, or White. For ethnicity, students could indicate whether they were Hispanic, Latino, Latina, or of Spanish origin. Questions used in this study are listed in the eAppendix in the Supplement.

### Outcomes and Measures

#### Current Use

Current use of e-cigarettes was defined as self-reported use on 1 or more days during the past 30 days. Current exclusive e-cigarette use was defined as current use of e-cigarettes but no other tobacco product (cigarettes, cigars [cigars, cigarillos, or little cigars], smokeless tobacco [chewing tobacco, snuff, dip, snus, or dissolvable tobacco], hookah tobacco, pipe tobacco, bidis, or heated tobacco products) on 1 or more days during the past 30 days. Current e-cigarette and other tobacco product use was defined as use of e-cigarettes and at least 1 other tobacco product on 1 or more days during the past 30 days.

#### Frequency of Use

Frequency of e-cigarette use among current e-cigarette users was assessed by the question, “During the past 30 days, on how many days did you use e-cigarettes?” Consistent with previous analyses,^8,18^ responses were categorized as 1 to 5 days, 6 to 19 days, and 20 to 30 days. Daily use (all 30 days during the past 30 days) was also examined.

#### Usual Brand

Usual brand was assessed differently compared with previous years.^7^ All current e-cigarette users were first asked, “During the past 30 days, what e-cigarette brands did you use? (Select one or more).” Response options were as follows: blu, JUUL, Logic, NJOY, SMOK, Suorin, Vuse, “some other brand(s) not listed here” (write-in responses available), and “I don’t know the brand.” Those who selected more than 1 option were then asked, “During the past 30 days, what brand of e-cigarettes did you usually use? (Choose only one answer).” The same response options as the first question were available with the additional response option of “I did not use a usual brand.” If a single brand was selected in the first question, that brand was reported as their usual brand. Otherwise, the option selected in the second question was recorded as the usual brand. Write-in responses for Puff Bar and those corresponding to an original response option were recoded.

#### Access Source

Sources of e-cigarette access were assessed by the question, “During the past 30 days, where did you get or buy the e-cigarettes that you have used? (Select one or more).” Response options were as follows: “a gas station or convenience store,” “a grocery store,” “a drugstore,” “a mall or shopping center kiosk/stand,” “on the internet,” “a vape shop or other store that only sells e-cigarettes,” “from a family member,” “from a friend,” “from some other person that is not a family member or a friend,” and “some other place not listed here” (write-in responses available but not assessed).

#### Flavors

Flavored e-cigarette use was assessed differently compared with previous years.^7^ All current e-cigarette users were asked, “Were any of the e-cigarettes that you used in the past 30 days flavored to taste like menthol, mint, clove or spice, alcohol (wine, cognac), candy, fruit, chocolate, or any other flavor?” (response options were “yes,” “no,” or “don’t know”). Those who responded yes were then asked, “What flavors were the e-cigarettes that you have used in the past 30 days? (Select one or more).” Response options were “menthol,” “mint,” “clove or spice,” “alcoholic drinks (such as wine, cognac, margarita, or other cocktails),” “candy, desserts, or other sweets,” “fruit,” “chocolate,” and “some other flavor not listed here” (write-in responses available but not assessed).

In addition, flavor types used in e-cigarettes were assessed by self-reported e-cigarette device type. All current e-cigarette users were asked, “Which of the following best describes the type of e-cigarette you have used in the past 30 days? If you have used more than one type, please think about the one you use most often.” Response options were as follows: “a disposable e-cigarette that uses pre-filled pods or cartridges (eg, JUUL),” “a disposable e-cigarette,” “an e-cigarette with a tank that you refill with liquids,” “a mod system (an e-cigarette that can be customized by the user with their own combination of batteries or other parts),” and “I don’t know the type.”

### Statistical Analysis

Analyses were conducted using SAS-callable SUDAAN, version 11.0.3.^19^ Data were weighted to account for the complex survey design and adjusted for nonresponse and varying selection probabilities. Weighted, nationally representative prevalence estimates with 95% CIs were separately computed among HS and MS students. Statistical comparisons (2-tailed, unpaired *t* tests) with 2019 data^17^ were conducted for measures that were assessed similarly in 2020 (see eAppendix in the Supplement). For all analyses, 2-sided *P* < .05 was considered statistically significant. Estimates were considered statistically unreliable and were suppressed if the unweighted denominator was less than 50 or the relative SE was greater than 30%.

## Results

Among the 14 531 respondents, grade levels were evenly distributed among HS and MS students. The weighted distribution of sex varied by school level (HS: 49.9% [95% CI, 47.8%-52.0%] female students and 50.1% [95% CI, 48.0%-52.2%] male students; MS: 48.7% [95% CI, 47.7%-49.8%] female students and 51.3% [95% CI, 50.2%-52.3%] male students). The weighted distribution of race/ethnicity was highest for non-Hispanic White students (HS: 55.7% [95% CI, 49.7%-61.6%]; MS: 50.2% [95% CI, 43.3%-57.2%]) and lowest for non-Hispanic students of other races (HS: 6.4% [95% CI, 4.6%-8.9%]; MS: 9.5% [95% CI, 5.6%-15.7%]) (Table 1).

**Table 1.** Characteristics of Middle and High School Students, National Youth Tobacco Survey, 2020

Characteristic^a^	High school		Middle school	
	Unweighted No.	Weighted % (95% CI)	Unweighted No.	Weighted % (95% CI)
Sex^b^				
Female	3849	49.9 (47.8-52.0)	3481	48.7 (47.7-49.8)
Male	3594	50.1 (48.0-52.2)	3539	51.3 (50.2-52.3)
Race/ethnicity^c^				
Non-Hispanic White	4037	55.7 (49.7-61.6)	3025	50.2 (43.3-57.2)
Non-Hispanic Black	670	12.0 (9.1-15.6)	930	12.9 (10.0-16.5)
Hispanic	2255	25.9 (21.2-31.2)	2087	27.3 (21.5-33.9)
Non-Hispanic other race	428	6.4 (4.6-8.9)	709	9.5 (5.6-15.7)
Grade				
6th	NA	NA	2352	33.7 (29.4-38.2)
7th	NA	NA	2354	33.2 (30.7-35.7)
8th	NA	NA	2336	33.2 (31.0-35.4)
9th	1966	26.7 (24.9-28.6)	NA	NA
10th	1882	25.5 (24.2-26.8)	NA	NA
11th	1799	24.3 (22.8-26.0)	NA	NA
12th	1806	23.4 (22.1-24.9)	NA	NA

Abbreviation: NA, not applicable.

^a^ Data were collected from January 16, 2020, through March 16, 2020, among 14 531 respondents. School-level data exclude 36 respondents missing self-reported grade level, including response of “ungraded or other grade.”

^b^ Excludes 32 respondents (10 high school and 22 middle school respondents) missing self-reported sex.

^c^ Excludes 354 respondents (63 high school and 291 middle school respondents) missing self-reported race/ethnicity. Hispanic respondents could be of any race (White, Black or African American, or other race [American Indian or Alaska Native, Asian, Native Hawaiian or other Pacific Islander]).

### Current Use

In 2020, 19.6% (95% CI, 17.2%-22.2%) of HS students and 4.7% (95% CI, 3.6%-6.0%) of MS students were current e-cigarette users. Among current e-cigarette users, 63.2% (95% CI, 58.4%-67.6%) of HS users and 51.0% (95% CI, 44.6%-57.4%) of MS users were current exclusive users of e-cigarettes (Table 2). Current e-cigarette use significantly decreased during 2019 to 2020 from 27.5% to 19.6% (relative percent change [RPC], −28.7%) among HS students and from 10.5% to 4.7% (RPC, −55.2%) among MS students (eTable in the Supplement).

**Table 2.** Prevalence of Current (Past 30-Day) e-Cigarette Use and Associated e-Cigarette Use Characteristics, National Youth Tobacco Survey, 2020

	High school		Middle school	
	Unweighted No.	Weighted % (95% CI)	Unweighted No.	Weighted % (95% CI)
**Among all students**				
Current use of e-cigarettes^a^	1448	19.6 (17.2-22.2)	316	4.7 (3.6-6.0)
**Among current e-cigarette users**				
Frequency of use, d				
1-5	605	41.5 (38.1-44.9)	193	59.5 (52.3-66.3)
6-19	275	19.7 (17.2-22.4)	64	20.5 (15.4-26.7)
20-30	568	38.9 (35.2-42.6)	59	20.0 (16.0-24.8)
Daily e-cigarette use^b^	340	22.5 (19.0-26.4)	26	9.4 (5.6-15.2)
Usual brand^c^				
Do not know	245	16.4 (13.8-19.4)	82	26.9 (18.2-37.9)
No usual brand	48	3.1 (2.3-4.3)	NA^d^	NA^d^
JUUL	347	25.4 (18.8-33.4)	120	35.1 (27.9-43.1)
NJOY	256	16.4 (11.2-23.3)	NA^d^	NA^d^
SMOK	127	8.5 (6.3-11.3)	NA^d^	NA^d^
Suorin	60	3.9 (2.2-6.8)	NA^d^	NA^d^
Vuse	45	3.2 (2.2-4.6)	15	6.1 (3.5-10.6)
blu	15	1.3 (0.7-2.4)	NA^d^	NA^d^
Logic	NA^d^	NA^d^	NA^d^	NA^d^
Puff Bar^e^	87	7.3 (4.3-12.4)	NA^d^	NA^d^
Some other brand	201	14.1 (10.0-19.7)	23	5.3 (3.3-8.2)
Access^f^				
A friend	793	57.1 (52.6-61.4)	173	58.9 (51.4-66.1)
Gas station or convenience store	314	22.2 (18.3-26.6)	40	13.7 (10.0-18.4)
Vape shop	254	17.5 (14.3-21.3)	28	9.1 (5.9-13.8)
Some other person (not family or friend)	249	17.0 (14.2-20.2)	51	17.7 (12.6-24.3)
A family member	131	8.4 (6.7-10.5)	75	27.6 (21.9-34.2)
On the internet	63	5.4 (4.1-7.1)	23	8.4 (5.4-12.9)
A drugstore	51	4.2 (2.8-6.2)	NA^d^	NA^d^
Mall or shopping center kiosk or stand	23	1.7 (1.0-2.7)	NA^d^	NA^d^
Grocery store	30	2.1 (1.2-3.9)	NA^d^	NA^d^
Some other place not listed here	55	4.2 (2.9-6.0)	13	3.6 (2.0-6.1)
Current exclusive use of e-cigarettes^g^	924	63.2 (58.4-67.6)	161	51.0 (44.6-57.4)
Current use of e-cigarettes and ≥1 other tobacco product^h^	524	36.8 (32.4-41.6)	155	49.0 (42.6-55.4)

Abbreviation: NA, not available.

^a^ Current use of e-cigarettes was defined as use of e-cigarettes on 1 or more days during the past 30 days.

^b^ Daily e-cigarette use was defined as use on all 30 days in the past 30 days in response to the question, “During the past 30 days, on how many days did you use e-cigarettes?”

^c^ Usual brand was determined by 2 questions. All current e-cigarette users were first asked, “During the past 30 days, what e-cigarette brands did you use? (Select one or more).” Respondents selecting more than 1 option were then asked, “During the past 30 days, what brand of e-cigarettes did you usually use? (Choose only one answer).” If a respondent only selected 1 response to the first question, that brand was recorded as their usual brand. Otherwise, the option selected in the second question was recorded as the usual brand.

^d^ Data are statistically unreliable because of an unweighted denominator less than 50 or relative SE greater than 30%.

^e^ Puff Bar, although not from a list of original response options, was the most commonly provided write-in response for “some other brand”; the write-in responses for Puff Bar were recoded. In addition, write-in responses corresponding to an original response option were recoded. All remaining responses were maintained as “some other brand.”

^f^ Sources of e-cigarette access among current e-cigarette users were assessed by the following: “During the past 30 days, where did you get or buy the e-cigarettes that you have used? (Select one or more).” Response options were “a gas station or convenience store,” “a grocery store,” “a drugstore,” “a mall or shopping center kiosk/stand,” “on the internet,” “a vape shop or other store that only sells e-cigarettes,” “from a family member,” “from a friend,” “from some other person that is not a family member or a friend,” and “some other place not listed here” (write-in responses available but not assessed).

^g^ Current exclusive e-cigarette use was defined as use of e-cigarettes but no other tobacco product (e-cigarettes, cigarettes, cigars [cigars, cigarillos, or little cigars], smokeless tobacco [chewing tobacco, snuff, dip, snus, or dissolvable tobacco], hookahs, pipe tobacco, bidis, or heated tobacco products) on 1 or more days during the past 30 days.

^h^ Current e-cigarette and 1 or more other tobacco product use was defined as use of e-cigarettes and at least 1 other tobacco product (cigarettes, cigars [cigars, cigarillos, or little cigars], smokeless tobacco [chewing tobacco, snuff, dip, snus, or dissolvable tobacco], hookahs, pipe tobacco, bidis, or heated tobacco products) on 1 or more days during the past 30 days.

### Frequency of Use

Among current exclusive e-cigarette users, 38.9% (95% CI, 35.2%-42.6%) of HS users and 20.0% (95% CI, 16.0%-24.8%) of MS users reported using e-cigarettes on 20 or more days in the past 30 days (Table 2). Daily e-cigarette use in the past 30 days was reported among 22.5% (95% CI, 19.0%-26.4%) of HS users and 9.4% (95% CI, 5.6%-15.2%) of MS users. Nondaily e-cigarette use was reported among 77.5% (95% CI, 73.6%-81.0%) of HS users and 90.6% (95% CI, 84.8%-94.4%) of MS users. During 2019 to 2020, the only frequency of use category to significantly change was e-cigarette use on 1 to 5 days in the past 30 days among HS users, which decreased from 46.4% to 41.5% (RPC, −10.6%) (eTable in the Supplement).

### Usual Brand

Among current e-cigarette users, JUUL was the most commonly reported usual brand (HS: 25.4%; 95% CI, 18.8%-33.4%; MS: 35.1%; 95% CI, 27.9%-43.1%) (Table 2). The disposable e-cigarette brand Puff Bar, reported as the usual brand among 7.3% (95% CI, 4.3%-12.4%) of HS users, was the most commonly provided write-in response for “some other brand.” Notably, 16.4% (95% CI, 13.8%-19.4%) of HS users and 26.9% (95% CI, 18.2%-37.9%) of MS users reported not knowing the e-cigarette brand they usually used (Table 2).

### Access Source

More than half of current e-cigarette users reported getting or buying the e-cigarettes they used during the past 30 days from a friend (HS: 57.1%; 95% CI, 52.6%-61.4%; MS: 58.9%; 95% CI, 51.4%-66.1%) (Table 2). The second most common source of e-cigarettes was gas stations and convenience stores among HS users (22.2%; 95% CI, 18.3%-26.6%) and a family member among MS users (27.6%; 95% CI, 21.9%-34.2%). During 2019 to 2020, changes were detected among MS users who reported “some other place not listed here,” which decreased from 8.4% to 3.6% (RPC, −57.1%) (eTable in the Supplement) and among HS users who reported getting or buying e-cigarettes from someone other than a family member or friend, which increased from 12.8% to 17.0% (RPC, 32.8%).

### Flavors

Among current exclusive e-cigarette users, an estimated 85.0% (95% CI, 82.2%-87.5%) of HS students and 71.9% (95% CI, 63.0%-79.3%) of MS students reported flavored e-cigarette use, which was consistent with the prevalence of flavored e-cigarette use among current users overall (Table 3). Fruit was the most commonly reported flavor type among current e-cigarette users, regardless of whether they used e-cigarettes exclusively or used multiple tobacco products.

**Table 3.** Flavored e-Cigarette Use and Flavor Types Among Current e-Cigarette Users, National Youth Tobacco Survey, 2020

	High school		Middle school	
	Unweighted No.	Weighted % (95% CI)	Unweighted No.	Weighted % (95% CI)
**Among current e-cigarette users**^**a**^				
Flavored e-cigarette use^b^				
Yes	1226	84.7 (82.2-86.9)	229	73.9 (66.9-79.8)
No	165	12.3 (10.4-14.6)	52	16.5 (10.9-24.1)
Do not know	44	2.9 (2.0-4.3)	27	9.7 (6.2-14.8)
Flavor types used^c^				
Fruit	886	73.1 (69.1-76.8)	163	75.6 (70.5-80.2)
Mint	697	55.8 (51.7-59.8)	108	46.5 (38.1-55.2)
Menthol	439	37.0 (31.0-43.4)	46	23.5 (18.1-29.9)
Candy, desserts, or other sweets	447	36.4 (33.1-39.9)	104	47.2 (40.4-54.2)
Chocolate	45	3.6 (2.5-5.2)	31	14.9 (8.7-24.4)
Alcoholic drink	70	5.3 (4.0-7.1)	24	11.6 (6.3-20.4)
Clove or spice	NA^d^	NA^d^	NA^d^	NA^d^
Other flavor not listed	146	12.5 (10.4-14.9)	45	21.3 (14.9- 29.5)
**Among current exclusive e-cigarette users**^**e**^				
Flavored e-cigarette use^b^				
Yes	784	85.0 (82.2-87.5)	110	71.9 (63.0-79.3)
No	99	12.0 (9.5-15.0)	30	18.7 (11.5-29.1)
Do not know	30	3.0 (1.9-4.5)	NA^d^	NA^d^
Flavor types used^c^				
Fruit	572	75.5 (70.9-79.5)	75	75.6 (67.1-82.5)
Mint	429	53.5 (48.0-59.0)	50	43.6 (31.2-57.0)
Menthol	245	32.2 (25.5-39.6)	23	26.3 (17.5-37.5)
Candy, desserts, or other sweets	261	33.0 (28.7-37.7)	46	46.9 (36.0-58.1)
Chocolate	NA^d^	NA^d^	NA^d^	NA^d^
Alcoholic drink	28	3.3 (2.1-4.9)	NA^d^	NA^d^
Clove or spice	NA^d^	NA^d^	NA^d^	NA^d^
Other flavor not listed	80	11.1 (8.6-14.1)	19	19.9 (11.6-31.9)
**Among current users of e-cigarettes and ≥1 other tobacco product**^**f**^				
Flavored e-cigarette use^b^				
Yes	442	84.2 (79.6-87.9)	119	75.9 (66.2-83.5)
No	66	12.9 (9.6-17.1)	22	14.2 (8.0-23.9)
Do not know	NA^d^	NA^d^	NA^d^	NA^d^
Flavor types used^c^				
Fruit	314	69.1 (63.3-74.4)	88	75.6 (69.0-81.2)
Mint	268	59.7 (53.8-65.3)	58	49.3 (37.7-61.0)
Menthol	194	45.3 (38.6-52.1)	23	20.8 (15.4-27.4)
Candy, desserts, or other sweets	186	42.3 (37.8-46.9)	58	47.5 (36.7-58.5)
Chocolate	33	6.7 (4.8-9.4)	NA^d^	NA^d^
Alcoholic drink	42	8.9 (6.1-12.7)	19	15.8 (9.0-26.3)
Clove or spice	NA^d^	NA^d^	NA^d^	NA^d^
Other flavor not listed	66	14.9 (11.2-19.7)	26	22.6 (13.7-35.1)

Abbreviation: NA, not available.

^a^ Current e-cigarette use was defined as use of e-cigarettes on 1 or more days during the past 30 days.

^b^ Flavored e-cigarette use was assessed by the question, “Were any of the e-cigarettes that you used in the past 30 days flavored to taste like menthol, mint, clove or spice, alcohol (wine, cognac), candy, fruit, chocolate, or any other flavor?” (response options: yes, no, or don’t know).

^c^ Among current flavored e-cigarette users, flavor types were assessed by the question, “What flavors were the e-cigarettes that you have used in the past 30 days? (Select one or more).” Respondents could select 1 or more of the following: “menthol,” “mint,” “clove or spice,” “alcoholic drinks (such as wine, cognac, margarita, or other cocktails),” “candy, desserts, or other sweets,” “fruit,” “chocolate,” or “some other flavor not listed here” (write-in responses not reported).

^d^ Data are statistically unreliable because of an unweighted denominator less than 50 or relative SE greater than 30%.

^e^ Current exclusive e-cigarette use was defined as use of e-cigarettes but no other tobacco products (cigarettes, cigars [cigars, cigarillos, or little cigars], smokeless tobacco [chewing tobacco, snuff, dip, snus, or dissolvable tobacco], hookahs, pipe tobacco, bidis, or heated tobacco products) on 1 or more days during the past 30 days.

^f^ Current e-cigarette and 1 or more other tobacco product use was defined as use of e-cigarettes and at least 1 other tobacco product (cigarettes, cigars [cigars, cigarillos, or little cigars], smokeless tobacco [chewing tobacco, snuff, dip, snus, or dissolvable tobacco], hookahs, pipe tobacco, bidis, or heated tobacco products) on 1 or more days during the past 30 days.

Overall use of flavor types also varied among current flavored e-cigarette users by device type (Table 4). Among current exclusive e-cigarette users overall, fruit-flavored e-cigarettes were the most commonly reported flavor type among users of prefilled pods or cartridges (67.3%; 95% CI, 60.9%-73.0%), disposable e-cigarettes (85.8%; 95% CI, 79.8%-90.3%), and tank-based devices (82.7%; 95% CI, 68.9%-91.1%), followed by mint flavored e-cigarettes (Table 4 and Figure). Estimates by flavor and device type were predominantly unstable among MS exclusive flavored e-cigarette users (eFigure in the Supplement).

**Table 4.** Flavor Types Used Among Current (Past 30-Day) Flavored e-Cigarette Users by Device Type, National Youth Tobacco Survey, 2020

	Device type, weighted % (95% CI)^a^				
	Prefilled pods or cartridges	Disposables	Tanks	Mod systems	Do not know
**Among current flavored e-cigarette users**^**b**^					
Flavor types used^c^					
Fruit^d^	66.0 (60.7-70.9)	82.7 (77.7-86.8)	81.7 (72.3-88.4)	78.9 (66.5-87.6)	61.9 (48.5-73.8)
Mint	57.5 (53.0-61.9)	51.9 (45.5-58.3)	49.3 (40.6-58.1)	63.3 (49.9-74.8)	46.5 (33.6-59.9)
Candy, desserts, other sweets^d^	35.6 (30.9-40.7)	41.7 (36.6-46.9)	39.2 (31.3-47.7)	57.4 (42.0-71.5)	19.9 (11.5-32.2)
Menthol^d^	44.5 (38.0-51.2)	23.3 (17.6-30.2)	31.7 (23.9-40.6)	29.6 (16.9-46.5)	26.8 (15.4-42.6)
Chocolate^d^	3.9 (2.7-5.6)	NA^e^	7.3 (4.5-11.6)	NA^e^	NA^e^
Clove or spice	NA^e^	NA^e^	NA^e^	NA^e^	NA^e^
Alcoholic drink^d^	6.6 (4.5-9.5)	4.1 (2.3-7.3)	8.2 (4.8-13.5)	NA^e^	NA^e^
Some other flavor^d^	15.9 (12.2-20.5)	11.4 (7.5-17.0)	10.9 (7.0-16.6)	22.7 (12.5-37.5)	NA^e^
**Among current exclusive e-cigarette users who used flavored e-cigarettes**^**f**^					
Flavor types used^c^					
Fruit^d^	67.3 (60.9-73.0)	85.8 (79.8-90.3)	82.7 (68.9-91.1)	NA^e^	NA^e^
Mint	56.0 (49.7-62.1)	50.0 (41.7-58.3)	47.0 (33.7-60.8)	NA^e^	NA^e^
Candy, desserts, other sweets	33.6 (27.8-40.0)	35.8 (29.0-43.1)	38.2 (28.7-48.8)	NA^e^	NA^e^
Menthol^d^	42.1 (34.3-50.3)	18.1 (12.4-25.6)	27.6 (18.2-39.6)	NA^e^	NA^e^
Chocolate	NA^e^	NA^e^	NA^e^	NA^e^	NA^e^
Clove or spice	NA^e^	NA^e^	NA^e^	NA^e^	NA^e^
Alcoholic drink	5.1 (3.0-8.4)	NA^e^	NA^e^	NA^e^	NA^e^
Some other flavor^d^	15.3 (11.2-20.5)	8.0 (4.7-13.3)	NA^e^	NA^e^	NA^e^
**Among current users of e-cigarettes and ≥1 other tobacco product who used flavored e-cigarettes**^**g**^					
Flavor types used^c^					
Fruit^d^	63.9 (56.0-71.1)	76.1 (67.0-83.3)	80.4 (69.8-88.0)	NA^e^	NA^e^
Mint	60.0 (53.4-66.1)	55.9 (45.7-65.6)	52.4 (39.8-64.6)	NA^e^	NA^e^
Candy, desserts, other sweets^d^	38.9 (33.5-44.5)	54.1 (45.6-62.4)	40.4 (30.1-51.7)	NA^e^	NA^e^
Menthol^d^	48.4 (41.5-55.3)	34.3 (24.6-45.6)	37.0 (26.4-49.2)	NA^e^	NA^e^
Chocolate	6.1 (4.0-9.3)	NA^e^	10.3 (5.7-17.8)	NA^e^	NA^e^
Clove or spice	NA^e^	NA^e^	NA^e^	NA^e^	NA^e^
Alcoholic drink	9.0 (5.4-14.7)	NA^e^	NA^e^	NA^e^	NA^e^
Some other flavor	16.9 (12.0-23.4)	18.5 (11.0-29.5)	NA^e^	NA^e^	NA^e^

Abbreviation: NA, not available.

^a^ Device type among current e-cigarette users was assessed by the question, “Which of the following best describes the type of e-cigarette you have used in the past 30 days? If you have used more than one type, please think about the one you use most often.” Response options included the following: “an e-cigarette that uses prefilled pods or cartridges (eg, JUUL),” “a disposable e-cigarette,” “an e-cigarette with a tank that you refill with liquids,” “a mod system (an e-cigarette that can be customized by the user with their own combination of batteries or other parts),” and “I don’t know the type.”

^b^ Current e-cigarette use was defined as use of e-cigarettes on 1 or more days during the past 30 days.

^c^ Flavored e-cigarette users refer to current e-cigarette users who answered “yes” to the question, “Were any of the e-cigarettes that you used in the past 30 days flavored to taste like menthol, mint, clove or spice, alcohol (wine, cognac), candy, fruit, chocolate, or any other flavor?” Flavor types among current e-cigarette users were assessed by the question, “What flavors were the e-cigarettes that you have used in the past 30 days? (Select one or more).” Respondents could select 1 or more of the following: “menthol,” “mint,” “clove or spice,” “alcoholic drinks (such as wine, cognac, margarita, or other cocktails),” “candy, desserts, or other sweets,” “fruit,” “chocolate,” or “some other flavor not listed here” (write-in responses not available but not assessed).

^d^ Statistically significant difference across device types (χ^2^ test *P* < .05).

^e^ Data are statistically unreliable because of an unweighted denominator less than 50 or relative SE greater than 30%.

^f^ Current exclusive e-cigarette use was defined as use of e-cigarettes but no other tobacco product(s) (cigarettes, cigars [cigars, cigarillos, or little cigars], smokeless tobacco [chewing tobacco, snuff, dip, snus, or dissolvable tobacco], hookahs, pipe tobacco, bidis, or heated tobacco products) on 1 or more days during the past 30 days.

^g^ Current e-cigarette and other tobacco product use was defined as use of e-cigarettes and 1 or more other tobacco product (cigarettes, cigars [cigars, cigarillos, or little cigars], smokeless tobacco [chewing tobacco, snuff, dip, snus, or dissolvable tobacco], hookahs, pipe tobacco, bidis, or heated tobacco products) on 1 or more days during the past 30 days.

**Figure.**
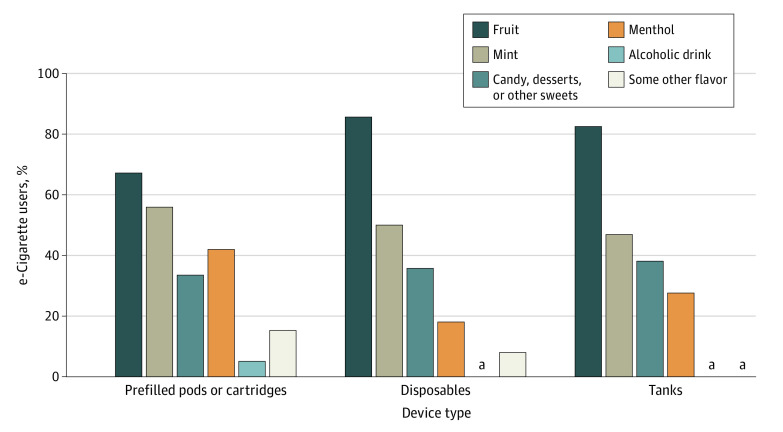
Flavor Types Used Among Current (Past 30-Day) Exclusive e-Cigarette Users who Reported Flavored e-Cigarette Use by Device Type, National Youth Tobacco Survey, 2020 For each cluster (individual device type), bars show weighted prevalence estimates of individual flavor types reported among current exclusive users of flavored e-cigarettes. Use of fruit, menthol, or some other flavor significantly differed by device types (χ^2^ test *P* < .05). ^a^Results not shown because of statistically unreliable estimates (unweighted denominator <50 or relative SE >30%). Estimates for mod systems, those who reported “I don’t know the type” for device type and the flavor types “chocolate” and “clove or spice” are also not shown because of statistically unreliable estimates.

## Discussion

In this survey study, approximately 1 in 5 HS students (19.6%) and 1 in 20 MS students (4.7%) reported current e-cigarette use in 2020. This finding reflects a decrease from 2019, which may be attributable to a variety of factors, including local-, state-, and national-level strategies to address e-cigarette use among youth.^6,11^ In December 2019, the federal minimum age of sale of tobacco products increased from 18 to 21 years,^20^ and effective February 2020, the US Food and Drug Administration (FDA) under authority of the 2009 Family Smoking Prevention and Tobacco Control Act^21^ implemented prioritized enforcement against certain flavored e-cigarette products that appeal to youth, including fruit and mint flavors.^22^ Among other factors, the FDA’s expanded youth e-cigarette prevention public education campaign^23^ and widespread public health efforts to address the multistate outbreak of e-cigarette or vaping product use–associated lung injury (EVALI) may also have contributed to the observed decrease in e-cigarette use among youth.^24^ Similarly, current use of any tobacco product decreased by nearly half among MS students (RPC, −46.4%) and one-quarter (RPC, −24.4%) among HS students during 2019 to 2020.^16^ Nevertheless, with the assumption that the prevalence estimates from this survey are nationally representative and could be used to project to national population totals for US HS and MS students, an estimated 3.6 million youth reported current e-cigarette use in 2020. Therefore, continued actions are warranted to ensure sustained progress in preventing and reducing e-cigarette use among US youth.

An increasing body of evidence demonstrates that flavors are a strong driver for e-cigarette use among youth. Flavors are a primary reason youth report using e-cigarettes; most youth e-cigarette users first initiated use with a flavored product, and most youth e-cigarette users have previously reported using flavored products.^11,25,26,27,28,29^ The current study underscores the continued high prevalence of flavored use among current youth e-cigarette users, including an estimated 84.7% of HS students (2.53 million) and 73.9% of MS students (400 000) in 2020. Among HS students who were current e-cigarette users, approximately three-quarters (73.1%) reported using fruit-flavored e-cigarettes, whereas more than half (55.8%) reported using mint-flavored e-cigarettes, and more than one-third (37.0%) reported using menthol-flavored e-cigarettes.

Further examination of flavored e-cigarette use by device type is also informative because of recent changes in e-cigarette sales and regulations, including JUUL’s decision to remove the sale of nontobacco, nonmenthol flavors^30^ and the FDA’s priority enforcement against the manufacturing, distribution, and sale of unauthorized flavored, cartridge-based e-cigarettes other than tobacco or menthol flavored.^22^ Although a subset of the 2020 NYTS data may reflect e-cigarette use behaviors before the priority enforcement took effect on February 6, 2020, this study’s findings suggest prominent use of menthol e-cigarette use among US youth. For example, among current flavored e-cigarette users who also used at least 1 other tobacco product, menthol use ranged from approximately one-third (34.3%) of flavored disposable e-cigarette users to nearly half (48.4%) of flavored prefilled pod or cartridge users. Of importance, the 2020 NYTS inquired about mint and menthol as separate flavor types for the first time, and respondents could indicate the use of multiple flavor types. This approach differs from the 2020 Monitoring the Future survey,^31^ wherein respondents reported the e-cigarette flavor they used most often. It is possible that some youth may perceive mint and menthol interchangeably or may not fully comprehend the distinction between the 2 flavor types.

Consistent with its prominence in e-cigarette retail sales since 2017,^12,13^ JUUL was the most commonly reported usual brand among youth e-cigarette users. In addition, the emergence of disposable brands, including Puff Bar, is consistent with data indicating that disposable e-cigarettes are increasing in popularity.^32,33^ Of note, because Puff Bar estimates from this study were identified from respondent write-ins instead of a prespecified option, it is possible that the prevalence of use captured represents underestimates of use. Because disposable e-cigarettes remain available in youth-appealing flavors, this observed shift in use of disposable e-cigarettes among youth in 2020 may be the result of youth seeking flavors they like. In July 2020, the FDA notified 10 companies who did not have the required premarket authorization, including the manufacturers of Puff Bar, to remove their flavored disposable e-cigarettes and youth-appealing e-liquid products.^34^ Notably, in some instances, the Family Smoking Prevention and Tobacco Control Act does not preempt states and localities from implementing more restrictive regulations than the federal standard. For example, several communities have restricted flavored tobacco product sales, including e-cigarettes and menthol-flavored products.^35^

### Limitations

This study has limitations. First, a truncated data collection period occurred because of the COVID-19 pandemic, resulting in a lower school-level participation rate (49.9%) compared with prior years. Despite this, the 2020 NYTS student participation rate (87.4%) was high, and the truncated timeline did not affect the original survey design or the national representativeness of the weighted estimates among students in grades 6 to 12 who attended public and private schools. Second, because of the survey fielding period, data on e-cigarette use behavior could not be examined before and after FDA’s priority enforcement guidance went into effect. Third, direct comparisons of flavored tobacco product use and usual e-cigarette brand estimates with prior years were not conducted because these measures were assessed differently in 2020 to keep up with the rapidly changing tobacco product landscape. Fourth, data were self-reported and might be subject to recall and response bias. Fifth, the underlying assumptions used for the population estimates should be considered in interpreting the data for projections to national estimates of e-cigarette use. Sixth, these findings might not be generalizable to youth who are homeschooled, have dropped out of school, are in detention centers, or are enrolled in alternative schools.

## Conclusions

This study found decreased rates of e-cigarette use among HS and MS students; however, nearly 20% of HS students and nearly 5% of MS students used these tobacco products, and frequent use and flavored use remained high in 2020. These findings reinforce the importance of population-based strategies, coupled with FDA regulation, to reverse the youth e-cigarette epidemic and foster a tobacco-free generation. Additional strategies to reduce tobacco product use and initiation among youth include increasing prices of tobacco products, protecting persons from exposure to secondhand smoke and aerosol from e-cigarettes, sustaining hard-hitting media campaigns that warn about the dangers of tobacco product use, and restricting young people’s access to tobacco products.^6,11^

## Notes

Supplement. **eTable.** Current (Past 30-Day) e-Cigarette Use, Frequency of e-Cigarette Use and e-Cigarette Access—National Youth Tobacco Survey, 2019-2020 **eFigure.** Flavor Types Used Among Middle School and High School Current (Past 30-Day) Exclusive Flavored e-Cigarette Users, by Device Type—National Youth Tobacco Survey, 2020 **eAppendix.** 2020 National Youth Tobacco Survey Questions Used in This Study eReferences
