# Typographic Error in Results

**DOI:** 10.1001/jamanetworkopen.2021.20001

**Published:** 2021-06-25

In the Original Investigation titled “Characteristics of e-Cigarette Use Behaviors Among US Youth, 2020,”^1^ published June 7, 2021, a typographic error appeared in the Results section under Frequency of Use. The word “exclusive” has been deleted from the opening clause of the first sentence. The article has been corrected.^1^

## References

[1] Wang TW, Gentzke AS, Neff LJ, et al. Characteristics of e-cigarette use behaviors among US youth, 2020. *JAMA Netw Open*. 2021;4(6):e2111336. doi:10.1001/jamanetworkopen.2021.11336 PMC8185598 34097049

