# Supplementary material for: Characteristics of e-Cigarette Use Behaviors Among US Youth, 2020
Source: JAMA Netw Open. 2021 Jun 7;4(6):e2111336. doi: 10.1001/jamanetworkopen.2021.11336 (PMC8185598; doi:10.1001/jamanetworkopen.2021.11336)
Supplement: Supplement. — eTable. Current (Past 30-Day) e-Cigarette Use, Frequency of e-Cigarette Use and e-Cigarette Access—National Youth Tobacco Survey, 2019-2020 eFigure. Flavor Types Used Among Middle School and High School Current (Past 30-Day) Exclusive Flavored e-Cigarette Users, by Device Type—National Youth Tobacco Survey, 2020 eAppendix. 2020 National Youth Tobacco Survey Questions Used in This Study eReferences [file jamanetwopen-e2111336-s001.pdf]

Supplementary Online Content

Wang TW, Gentzke AS, Neff LJ, et al. Characteristics of e-cigarette use behaviors among US youth, 2020. *JAMA Netw Open*. 2021;4(6):e2111336. doi: 10.1001/jamanetworkopen.2021.11336

eTable. Current (Past 30-Day) e-Cigarette Use, Frequency of e-Cigarette Use and e-Cigarette Access—National Youth Tobacco Survey, 2019-2020

eFigure. Flavor Types Used Among Middle School and High School Current (Past 30-Day) Exclusive Flavored e-Cigarette Users, by Device Type—National Youth Tobacco Survey, 2020

eAppendix. 2020 National Youth Tobacco Survey Questions Used in This Study

eReferences

This supplementary material has been provided by the authors to give readers additional information about their work.

eTable. Current (Past 30-Day) e-Cigarette Use, Frequency of e-Cigarette Use and e-Cigarette Access — National Youth Tobacco Survey, 2019-2020

	2019[a]			2020[b]		2019-2020
	**Unweighted, no.**	**% (95% CI)**		**Unweighted, no.**	**% (95% CI)**	**Relative Percent Change (%)**
**Middle School**						
Current use of e-cigarettes[c]	902	10.5 (9.4-11.8)		316	**4.7 (3.6-6.0)[g]**	-55.2
**Frequency of use**, d **(among current e-cigarette users)**[d]						
1-5	590	65.3 (61.0-69.5)		193	59.5 (52.3-66.3)	-8.9
6-19	159	16.7 (13.5-20.4)		64	20.5 (15.4-26.7)	22.8
20-30	153	18.0 (15.2-21.2)		59	20.0 (16.0-24.8)	11.1
Daily e-cigarette use	80	8.8 (6.9-11.2)		26	9.4 (5.6-15.2)	6.8
**Access Source**[e] **(among current e-cigarette users)**						
A friend	538	60.5 (56.2-64.6)		173	58.9 (51.4-66.1)	-2.6
Gas station or convenience store	83	9.5 (7.0-12.7)		40	13.7 (10.0-18.4)	44.2
Vape shop	68	7.9 (5.8-10.7)		28	9.1 (5.9-13.8)	15.2
Some other person (not family/friend)	110	14.1 (10.9-18.1)		51	17.7 (12.6-24.3)	-25.5
A family member	184	21.8 (18.9-25.1)		75	27.6 (21.9-34.2)	26.6
On the internet	56	8.3 (6.1-11.2)		23	8.4 (5.4-12.9)	1.2
A drugstore	NA[f]	NA[f]		NA [f]	NA[f]	NA
Mall/shopping center kiosk or stand	19	1.6 (0.9-2.9)		NA [f]	NA[f]	NA
Grocery store	18	2.3 (1.3-4.0)		NA [f]	NA[f]	NA
Some other place not listed here	62	8.4 (5.7-12.4)		13	**3.6 (2.0-6.1)[g]**	-57.1
**High School**						
Current use of e-cigarettes	2,709	27.5 (25.3-29.7)		1,448	**19.6 (17.2-22.2)[g]**	-28.7
**Frequency of use**, d **(among current e-cigarette users)**						
1-5	1,266	46.4 (43.6-49.3)		605	**41.5 (38.1-44.9)[g]**	-10.6
6-19	526	19.4 (17.8-21.1)		275	19.7 (17.2-22.4)	1.5
20-30	917	34.2 (31.2-37.3)		568	38.9 (35.2-42.6)	13.7
Daily e-cigarette use	564	21.4 (19.0-24.0)		340	22.5 (19.0-26.4)	5.1
**Access (among current e-cigarette users)**						
A friend	1,587	59.1 (56.2-61.8)		793	57.1 (52.6-61.4)	-3.4
Gas station or convenience store	470	19.9 (17.4-22.6)		314	22.2 (18.3- 26.6)	11.6
Vape shop	450	17.4 (15.2-19.8)		254	17.5 (14.3-21.3)	0.6
Some other person (not family/friend)	316	12.8 (10.9-14.8)		249	**17.0 (14.2-20.2)[g]**	32.8
A family member	268	9.6 (8.4-11.1)		131	8.4 (6.7-10.5)	-12.5
On the internet	172	6.7 (5.3-8.3)		63	5.4 (4.1-7.1)	-19.4
A drugstore	81	3.0 (2.3-4.0)		51	4.2 (2.8-6.2)	40.0
Mall/shopping center kiosk or stand	67	3.0 (1.8-4.8)		23	1.7 (1.0-2.7)	-43.3
Grocery store	60	2.3 (1.5-3.3)		30	2.1 (1.2-3.9)	-8.7

© 2021 Wang TW et al. *JAMA Network Open.*

Some other place not listed here	140	5.2 (4.2-6.4)		55	4.2 (2.9-6.0)	-19.2

Abbreviation: NA, not available.

[a] Data collection for the 2019 National Youth Tobacco Survey occurred from February 15, 2019 to May 24, 2019 among 19,018 respondents.[1-3]

[b] Data collection for the 2020 National Youth Tobacco Survey occurred from January 16, 2020 to March 16, 2020 among 14,531 respondents.

[c] Current e-cigarette use was defined as use of e-cigarettes on 1 or more days during the past 30 days.

[d] Frequency of e-cigarette use among current e-cigarette users was assessed by: "During the past 30 days, on how many days did you use e-cigarettes"? Responses were categorized as 1-5 days, 6-19 days, and 20-30 days. Daily use (all 30 days during the past 30 days) was also examined.

[e] Sources of e-cigarette access among current e-cigarette users were assessed by: "During the past 30 days, where did you get or buy the e-cigarettes that you have used? (Select one or more)". Response options were: "a gas station or convenience store", "a grocery store", "a drugstore", "a mall or shopping center kiosk/stand" , "on the internet", "a vape shop or other store that only sells e-cigarettes", "from a family member", "from a friend", "from some other person that is not a family member or a friend", and "some other place not listed here" (write-in responses available but not assessed).

[f] Data are statistically unreliable because of an unweighted denominator less than 50 or relative SE greater than 30%.

[g] Weighted estimate in 2020 is significantly different from its corresponding weighted estimate in 2019 (t-test; p<0.05).

© 2021 Wang TW et al. *JAMA Network Open.*

eFigure. Flavor Types[a] Used among Middle School and High School Current (Past 30-Day) Exclusive Flavored e-Cigarette Users[b], by Device Type[c] — National Youth Tobacco Survey, 2020

(A) Flavor types used among middle school current exclusive flavored e-cigarette users, by device type

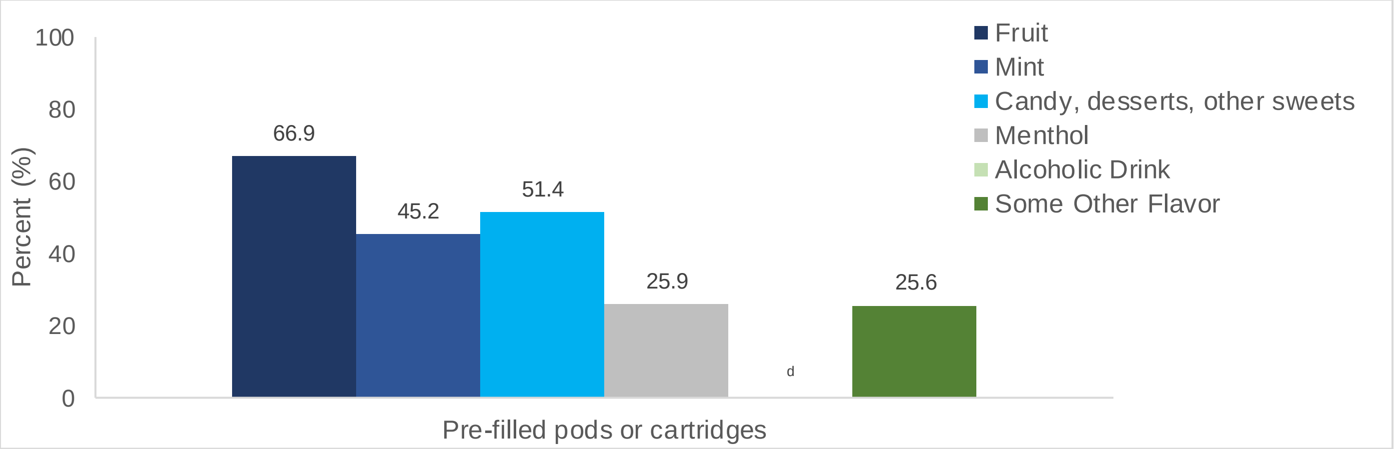

(B) Flavor types used among high school current exclusive flavored e-cigarette users, by device type

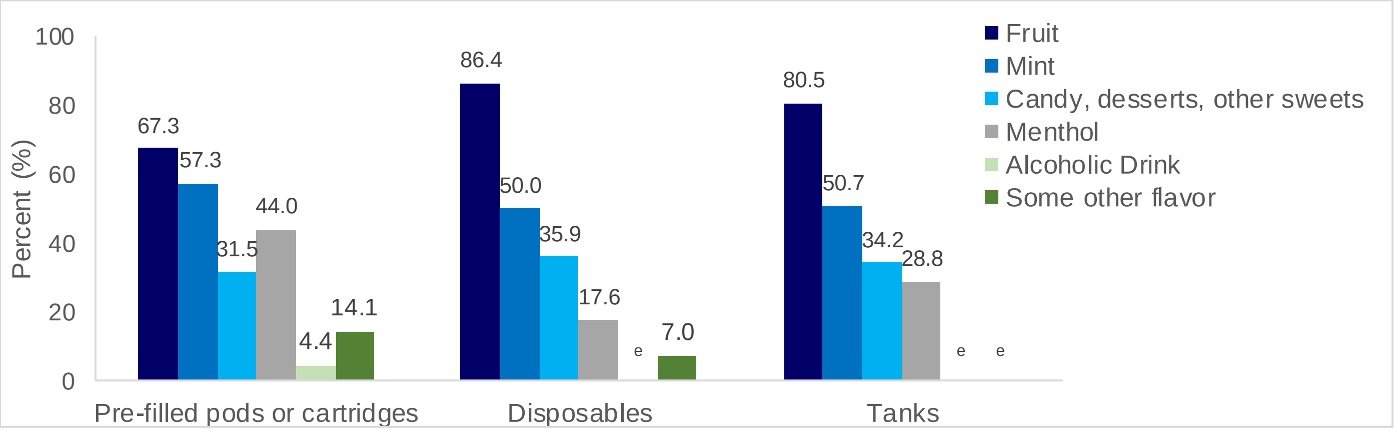

[a] Flavored e-cigarette users refer to current e-cigarette users who answered "yes" to the question, "Were any of the e-cigarettes that you used in the past 30 days flavored to taste like menthol, mint, clove or spice, alcohol (wine, cognac), candy, fruit, chocolate, or any other flavor?". Flavor types among flavored e-cigarette users were assessed by the question, "What flavors were the e-cigarettes that you have used in the past 30 days? (Select one or more)". Respondents could select 1 or more of the following: "menthol"; "mint"; "clove or spice"; "alcoholic drinks (such as wine, cognac, margarita, or other cocktails)"; "candy, desserts, or other sweets"; "fruit"; "chocolate"; or "some other flavor not listed here" (write-in responses available but not assessed).

[b] Current exclusive e-cigarette use was defined as use of e-cigarettes but no other tobacco products (cigarettes, cigars [cigars, cigarillos, or little cigars], smokeless tobacco [chewing tobacco, snuff, dip, snus, or dissolvable tobacco], hookahs, pipe tobacco, bidis, or heated tobacco products) on 1 or more days during the past 30 days.

[c] Device type among current e-cigarette users was assessed by the question, "Which of the following best describes the type of e-cigarette you have used in the past 30 days? If you have used more than one type, please think about the one you use most often". Response options included: "a disposable e-cigarette", "an e-cigarette that uses pre-filled pods or cartridges (e.g., JUUL)", "an e-cigarette with a tank that you refill with liquids", "a mod system (an e-cigarette that can be customized by the user with their own combination of batteries or other parts)," and "I don't know the type".

[d] Data are statistically unreliable because of an unweighted denominator less than 50 or relative SE greater than 30%. Estimates for disposables, mod systems, those who reported "I don't know the type" for device type, and flavor types "chocolate" and "clove or spice" also not shown because of statistically unreliable estimates.

[e] Data are statistically unreliable because of an unweighted denominator less than 50 or relative SE greater than 30%. Estimates for mod systems, those who reported "I don't know the type" for device type, and flavor types "chocolate" and "clove or spice" also not shown because of statistically unreliable estimates.

© 2021 Wang TW et al. *JAMA Network Open.*

eAppendix. 2020 National Youth Tobacco Survey Questions Used in This Study [a]

3. What grade are you in?
   - A. 6th
   - B. 7th
   - C. 8th
   - D. 9th
   - E. 10th
   - F. 11th
   - G. 12th
   - H. Ungraded or other grade

4. Are you Hispanic, Latino, Latina, or of Spanish origin? **(Select one or more)**
   - A. No, not of Hispanic, Latino, Latina, or Spanish origin
   - B. Yes, Mexican, Mexican American, Chicano, or Chicana
   - C. Yes, Puerto Rican
   - D. Yes, Cuban
   - E. Yes, Another Hispanic, Latino, Latina, or Spanish origin

5. What race or races do you consider yourself to be? **(Select one or more)**
   - A. American Indian or Alaska Native
   - B. Asian
   - C. Black or African American
   - D. Native Hawaiian or Other Pacific Islander
   - E. White

INSTRUCT_2. The next seven sections of questions ask about your use of particular kinds of tobacco products, such as e-cigarettes, cigarettes, cigars, smokeless tobacco, hookahs, roll-your-own cigarettes, pipes, snus, dissolvable tobacco, bidis, and heated tobacco products.

INSTRUCT_3. The next several questions are about electronic cigarettes or e-cigarettes, such as JUUL, Vuse, blu, and Logic. E-cigarettes are battery powered devices that usually contain a nicotine-based liquid that is vaporized and inhaled. You may also know them as e-cigs, vape-pens, e-hookahs, vapes, or mods.

6. Have you **ever used** an e-cigarette, even once or twice?
   - A. Yes
   - B. No

9. During the **past 30 days**, on how many days did you use e-cigarettes?

   Specify: |__|__| (Range 0 – 30)

11. Which of the following best describes the type of e-cigarette you have used in the **past 30 days**? If you have used more than one type, please think about the one you use most often.
   - A. A disposable e-cigarette
   - B. An e-cigarette that uses pre-filled pods or cartridges (e.g. JUUL)
   - C. An e-cigarette with a tank that you refill with liquids
   - D. A mod system (an e-cigarette that can be customized by the user with their own combination of batteries or other parts)
   - E. I don't know the type

© 2021 Wang TW et al. *JAMA Network Open.*

12. During the **past 30 days**, what e-cigarette brands did you use? **(Select one or more)**

A. blu
B. JUUL
C. Logic
D. NJOY
E. SMOK
F. Suorin
G. Vuse
H. Some other brand(s) not listed here (specify): ________________________________
I. I don't know the brand

13. During the **past 30 days**, what brand of e-cigarettes did you usually use? **(Choose only one answer)**

A. I did not use a usual brand
B. blu
C. JUUL
D. Logic
E. NJOY
F. SMOK
G. Suorin
H. Vuse
I. Some other brand not listed here (specify): ______________________________
J. I don't know the brand

15. During the **past 30 days**, where did you get or buy the e-cigarettes that you have used? (**Select one or more**)

A. A gas station or convenience store
B. A grocery store
C. A drugstore
D. A mall or shopping center kiosk/stand
E. On the Internet
F. A vape shop or other store that only sells e-cigarettes
G. Some other place not listed here (Specify: _________________________)
H. From a family member
I. From a friend
J. From some other person that is not a family member or a friend

INSTRUCT_4. The next several questions are about smoking cigarettes (ones that have to be lit and burned).

25. During the **past 30 days**, on how many days did you smoke cigarettes?

Specify: |__|__| (Range: 0 – 30)

INSTRUCT_5. The next several questions are about the use of cigars, cigarillos, or little cigars such as Swisher Sweets, Black and Mild, Garcia y Vega, Cheyenne, White Owl, or Dutch Masters.

37. During the **past 30 days**, on how many days did you smoke cigars, cigarillos, or little cigars?

Specify: |__|__| (Range 0 – 30)

INSTRUCT_6. The next several questions are about the use of chewing tobacco, snuff, or dip, such as Copenhagen, Grizzly, Skoal, or Longhorn. Do not think about snus or dissolvable tobacco products when you answer these questions
.

© 2021 Wang TW et al. *JAMA Network Open.*

46. During the **past 30 days**, on how many days did you use chewing tobacco, snuff, or dip?

Specify: |__|__| (Range 0 – 30)

INSTRUCT_7. The next several questions are about smoking tobacco in a hookah, which is a type of waterpipe. Shisha (or hookah tobacco) is smoked in a hookah.

50. During the **past 30 days**, on how many days did you smoke tobacco in a hookah or waterpipe?

Specify: |__|__| (Range 0 – 30)

INSTRUCT_8. The next section is about the use of other tobacco products, not described in the previous sections.

54. In the **past 30 days**, which of the following products have you used on **at least one day**? (**Select one or more**)

A. Roll-your-own cigarettes
B. Pipes filled with tobacco (not hookah or waterpipe)
C. Snus, such as Camel, Marlboro, or General Snus
D. Dissolvable tobacco products such as Ariva, Stonewall, Camel orbs, Camel sticks, Marlboro sticks, or Camel strips
E. Bidis (small brown cigarettes wrapped in a leaf)
F. I have not used any of the products listed above in the past 30 days

INSTRUCT_9. The next section is about heated tobacco products. Some people refer to these products as "heat-not-burn" tobacco products. Heated tobacco products heat tobacco sticks or capsules to produce a vapor. They are different from e-cigarettes, which heat a liquid to produce a vapor. Some brands of heated tobacco products include iQOS, glo, and Eclipse.

57. During the **past 30 days**, on how many days did you use a heated tobacco product?

Specify: |__|__| (Range 0 – 30)

INSTRUCT_12. The next few questions ask about flavors in tobacco products.

59 [A]. Were any of the **[e-cigarettes]** that you used in the **past 30 days** flavored to taste like menthol, mint, clove or spice, alcohol (wine, cognac), candy, fruit, chocolate, or any other flavor?

A. Yes
B. No
C. Don't Know

60 [A]. What flavors were the **[tobname1]** that you have used in the **past 30 days**? **(Select one or more)**

A. Menthol
B. Mint
C. Clove or spice
D. Fruit
E. Chocolate
F. Alcoholic drinks (such as wine, cognac, margarita, or other cocktails)
G. Candy, desserts, or other sweets
H. Some other flavor not listed here (Specify: ______________________________)

[a]The full 2020 NYTS questionnaire can be accessed at https://www.cdc.gov/tobacco/data_statistics/surveys/nyts/index.htm.

© 2021 Wang TW et al. *JAMA Network Open.*

References

1. Centers for Disease Control and Prevention. National Youth Tobacco Survey (NYTS). National Youth Tobacco Survey. https://www.cdc.gov/tobacco/data_statistics/surveys/nyts/index.htm.

2. Cullen KA, Gentzke AS, Sawdey MD, Chang JT, Anic GM, Wang TW, Creamer MR, Jamal A, Ambrose BK, King BA. e-Cigarette use among youth in the United States, 2019. JAMA. 2019 Dec 3;322(21):2095-103.

3. Wang TW, Gentzke AS, Creamer MR, Cullen KA, Holder-Hayes E, Sawdey MD, Anic GM, Portnoy DB, Hu S, Homa DM, Jamal A. Tobacco product use and associated factors among middle and high school students—United States, 2019. MMWR Surveillance Summaries. 2019 Dec 6;68(12):1.

© 2021 Wang TW et al. *JAMA Network Open.*
